# Longitudinal study on the progression of muscle status among community-dwelling ambulatory older multiethnic Asians with type 2 diabetes mellitus

**DOI:** 10.1186/s12877-022-03098-0

**Published:** 2022-05-21

**Authors:** Ngiap Chuan Tan, Usha Sankari, Chiat Eng Ng, Yi Ling Eileen Koh

**Affiliations:** 1 SingHealth Polyclinics, 167 Jalan Bukit Merah Connection One Tower 5 #15-10, Singapore, 150167 Singapore; 2 Duke NUS Medical School, 8 College Road, Singapore, 169857 Singapore

**Keywords:** Muscle mass, Handgrip strength, Gait speed, Type-2 diabetes mellitus, Asians

## Abstract

**Background:**

Muscle health decline with age, but its deterioration in older persons with type-2 diabetes mellitus (T2DM) over time is not well-established. This study aimed to determine the change in muscle mass, handgrip strength and gait speed over time among community-dwelling ambulatory older multi-ethnic Asian patients with T2DM and their associated factors.

**Methods:**

Among 387 eligible patients aged 60–89 years who were recruited at baseline, 348 (89.9%) were reviewed at a public primary care clinic in Singapore in the subsequent 9 to 34 months. The change in their clinical and functional status, levels of physical activity and muscle status (mass, grip strength and gait speed based on the Asian Working Group for Sarcopenia criteria) were recorded and compared. Their physical activity levels were assessed using the Physical Activity Scale for the Elderly (PASE) and International Physical Activity Questionnaire (IPAQ). Their quality of life was evaluated based on the World Health Organization Quality of Life (WHOQOL) domains. Linear regression analysis was used to identify the factors associated with muscle health change.

**Results:**

The study population comprised men (52.9%), Chinese (69.3%), mean age of 68.4 ± SD5.6 years and had at least secondary education (76.4%). Their mean muscle mass significantly decreased by 0.03 ± SD0.06 kg/m^2^/month, mean handgrip strength by 0.06 ± SD0.26 kg/month and negligible change in gait speed of 0.002 ± SD0.01 m/sec/month. Their mean weight significantly decreased by 0.5 ± SD3.9 kg, waist and hip circumferences by 2.5 ± SD6cm and 3.2 ± SD5.1 cm respectively, with no change in BMI. Linear regression shows significant associations between muscle mass change and education level (β = 0.36,*p* = 0.012, 95% CI = 0.08–0.64), BMI (β = 0.11,*p* = < 0.001, 95% CI = 0.05–0.17), change in medication class (β = 0.39,*p* = < 0.001, 95% CI = 0.06–0.71) and review interval (β = − 0.003, *p* < 0.001, 95% CI = -0.004--0.002). Gait speed change was associated with singlehood (β = − 0.13,*p* = 0.029, 95% CI = -0.25--0.01) and WHOQOL physical health (β = 0.01,*p* = 0.024, 95% CI = 0.00–0.02) domain. No factor was associated with handgrip strength change.

**Conclusions:**

The study population with T2DM showed significant decline in their mean weight, waist and hip circumferences, mean muscle mass and mean grip strength but gait speed was unaffected. Muscle mass change was associated with education level, BMI and length of review interval. Handgrip strength change was not significantly correlated with any factor. Gait speed change was associated with singlehood and physical health.

## Background

Sarcopenia refers to the age-related loss of muscle mass and muscle function. The three parameters underpin muscle health of an individual. The Asian Working Group for Sarcopenia (AWGS 2014) defines sarcopenia as low muscle mass (defined as skeletal muscle index < 7 kg/m^2^ in males and < 5.7 kg/m^2^ in females) associated with either low muscle strength (defined as handgrip strength < 26 kg in males and < 18 kg in females) or low physical performance (defined as six-meter gait speed ≤0.8 m/s) or both [1].

Aging, underlying inflammation, endocrine dysfunction such as insulin resistance, nutritional deficiency and physical inactivity are risk factors for sarcopenia [2]. Insulin resistance leads to type-2 diabetes mellitus (T2DM), which is rising in prevalence in Asia, especially in regions and communities with aging populations [3]. Hence, older people with T2DM seem to be at higher risk of poor muscle health, who may be aggravated by their dietary deficiency and physical inactivity [4]. Little is known on the rate of progression of sarcopenia among these community-dwelling ambulatory older patients with T2DM [5]. A recent cross-sectional study has shown that 58% of the local older patients with T2DM had pre-sarcopenia and sarcopenia [6]. Their risk factors such as age, diabetic nephropathy, hip circumference and multi-morbidity were significantly associated with sarcopenia. However, a single indicator of glycemic control based on the glycated hemoglobin (HbA1c) was not associated with sarcopenia in the same study. Glycemic control can be dynamic in these ambulatory older patients, being affected by their personal lifestyle and behavior such as dietary intake and physical activities over time. The longitudinal effects of combined personal demographic, clinical and environmental factors on the muscle health status of these older persons with T2DM in the community is not well-established.

In Singapore, about one in nine adults of its multi-ethnic Asian population has T2DM, with higher prevalence among its minority Malay and Indian ethnic groups. Its T2DM prevalence is estimated to increase to 15% in 2050 [7]. The World Health Organization has reported its population having the third highest longevity in the world in 2018 [8]. With increasing number of aging patients with T2DM year on year, the effect of sarcopenia on its population will escalate, with significant impact expected on their morbidity and mortality.

The majority of these patients with T2DM are managed in public primary care clinics (polyclinics) on the island-state. Their continuity of care in these polyclinics present opportunities to identify risk factors associated with the progression of sarcopenia. Hence, this prospective study primarily aimed to determine the change in the muscle health status (muscle mass, grip strength and gait speed) in community dwelling, ambulatory, older multi-ethnic Asian patients with T2DM over a period of up to 34 months. The secondary aim was to identify factors which were associated with their deteriorating muscle health status over time. Understanding the progression of the muscle health and associated factors will facilitate the design of appropriate interventions to mitigate sarcopenia.

## Methods

### Study site and period

The baseline study was published in Fung FY et al. [6]. The study data was collected at a public polyclinic in the north-eastern side of Singapore during October 2017 to March 2018. About 30% of the clinic patient attendances are aged 65 years and above.

The recruited patients were reviewed and repeat clinical measurements were performed in the same polyclinic over the next 34 months. The variability in period of review was due to the COVID-19 pandemic, which disrupted regular review of patients at the study site.

### Study participants

The profile of the participants included Singapore citizens or permanent residents aged 60–89 years who were enrolled into the baseline study and had a diagnosis of T2DM for at least 1 year in the electronic health records (EMR). The participants could be on diet control, oral hypoglycemic agents, or a combination of oral hypoglycemic agents with insulin injections.

Potential participants with history of stroke, carpal tunnel syndrome, severe hip or knee osteoarthritis, dysarthria or dysphasia, hearing difficulties, use of walking aid, physical disabilities that affected hand-grip and/or walking, use of electronic implants such as pacemaker, and living in residential care facilities were excluded from the study. Those with cognitive impairment or any form of other disabilities, as documented in the EMR, which rendered them incapable of providing informed written consent, were also excluded.

### Sample size estimation

Based on the study by Norshafarina SK et al. [9], the prevalence of sarcopenia among seniors with T2DM is 59.8%, with a 5% precision and 95% confidence level, the sample size required for the study is estimated to be 370. A 5% buffer for incomplete data was factored in and the final sample size was 388.

### Recruitment and muscle health assessment

Potential subjects were screened for eligibility criteria at the polyclinic. They were provided information of the study using the approved documents, clarified on their queries and recruited after the research assistant obtained their informed written consent. Next, the subjects administered the study questionnaire, either by themselves or by the research assistant, to collect their demographic, lifestyle habits, socio-economic indicators and clinical information. Other information collected in the questionnaire included their level of physical activity using the validated International Physical Activity Questionnaire (IPAQ) and Physical Activity Scale for the Elderly (PASE), and their quality of life using the World Health Organization Quality of Life scale (WHOQOL) with its four domains.

### Questionnaire

The IPAQ assesses the level of physical activity across a comprehensive set of domains including: leisure time physical activity, domestic and gardening (yard) activities, work-related physical activity, transport-related physical activity. IPAQ scores are grouped according to low, moderate and high level of physical activity. If the IPAQ scores have changed from low to moderate, or moderate to high or low to high, it shows that a subject improves in level of physical activity from baseline to next time-point of assessment. Vice-versa, a subject who decreases in physical activity, the IPAQ has declined from moderate to low, high to moderate or high to low score. If the scores are similar, it means that subject has maintained the same physical activity level.

The PASE assesses the level of PA of community-dwelling older adults based on three domains: leisure time, household and work-related activities. The total score can be computed by multiplying the amount of time spent for each activity by the respective weights and summing over all activities [10]. PASE scores may range from zero to 400 or more, higher score indicates more time spent on physical activity.

The WHOQOL consists of 26 items with 4-domain scores that denote the individual’s perception of quality of life in each particular domain. The 4 domains include (1) physical health, (2) psychological, (3) social relationships, and (4) environment. The 4 domains are treated as continuous outcomes with each domain score converted to scores ranging between 4 and 20, in accordance with the first transformation method outlined in the WHOQOL-BREF scoring instructions [11]. The two stand-alone questions related to QOL rating and Satisfaction with Health were analyzed separately [11]. Higher scores denote higher quality of life.

Next, anthropometric and clinical measurements were carried out, including their weight, height, body mass index (BMI), waist circumference (WC), hip circumference (HC), systolic and diastolic blood pressures. Using the AVAMECH Model B100U device for measurement, participants can view their weight, height and BMI in a printed copy. Blood pressures were taken twice using the OMRON HEM-7280 T blood pressure monitor. The blood pressures were measured twice using the automatic blood pressure monitor (OMRON HEM-7280 T). WC and HC were determined using the same measuring tape. WC was measured at the approximate midpoint between the lower margin of the last palpable rib and the top of the iliac crest while HC was measured around the widest portion of the pelvis [12].

The definition of sarcopenia in this study was based on the AWGS 2014 diagnostic criteria [1]. The muscle health assessment was conducted in the following ways:

1. The bio-electrical impedance analysis machine (OMRON Body composition monitor, Model HBF-375) was used to measure body muscle mass. It is then computed into skeletal muscle index by dividing the body muscle mass by squared body height.
2. according to the American Society of Hand Therapists’ guidelines, handgrip strength was measured twice on each hand, with the participant seated with elbow flexed at ninety degrees, forearm in neutral position and wrist between 0 and 30 degrees of dorsiflexion and supported on a Table 3], using a dynamometer (JAMAR Plus Digital Hand Dynamometer #563213). The average handgrip strength of the dominant hand was used for analysis.
3. gait speed was computed by taking the average of two time measurements taken to walk a linear distance of 6-m at the usual walking speed. Both run-in and run-out phases of approximately 1 m were provided before and after the six-meter distance respectively. A digital stopwatch (CASIO Model 611Q24R) was used to measure the time taken.

### Outcomes

Based on the AWGS criteria [1], sarcopenia is diagnosed when the skeletal muscle index is < 7 kg/m^2^ in males and < 5.7 kg/m^2^ in females, with either low handgrip strength of < 26 kg in males and < 18 kg in females, or low physical performance of six-meter gait speed ≤0.8 m/s or both.

The participants’ latest glycemic control index (HbA1C) and fasting lipid profile (total cholesterol, high-density lipoprotein cholesterol [HDL], low-density lipoprotein cholesterol [LDL], triglycerides [TG]) were retrieved from their laboratory test results in the electronic medical records. Other information such as the duration of T2DM, presence of diabetic complications (any documented retinopathy, nephropathy, neuropathy, vasculopathy), co-morbidities (diagnosis list) and medications (electronic prescription) were also obtained. The research assistant recorded the data in a secured research database system known as Redcap, which is password protected and accessed only by the research team members.

### Longitudinal data collection

The patients were contacted by the research assistant at least 10 months later from the enrolment for repeat measurements of their anthropometry, clinical and sarcopenia related parameters at the study site. The research assistant made at least two phone calls to contact the subjects for the next review. The patients were considered dropouts if they became uncontactable or failed to show up despite the phone calls. Their latest laboratory results were again extracted from their electronic medical records during the review and documented in the Redcap research database. The latest HbA1c of the patients was compared to the glycemic control index on study enrolment. The same questionnaire was administered again to determine the change in the scores from the IPAQ, PASE and WHOQOL scales. All data from the baseline and follow up were audited, rectified and de-identified before being analyzed.

### Statistical analyses

Descriptive statistics were computed for the demographics and their physical activity and quality of life (QOL), and the change in muscle health status were compared against the demographics using Independent t-test, ANOVA for the categorical variables, and Spearman’s correlation for the continuous variables. The baseline and follow up clinical parameters were assessed using paired t-tests. The potentially significant factors (*p* < =0.2 from bivariate analysis) affecting muscle health status were selected to be included in the multiple linear regression to control for the confounding factors. Adjusted beta estimates and their confidence intervals were presented. A *p*-value of less than 0.05 is considered to be statistically significant. All analyses were done using IBM SPSS version 27.0.

### Ethics approval and funding

Each recruited patient received a supermarket voucher valued at SGD5 (USD3.80) at enrolment and the subsequent review. This study was approved by the SingHealth Centralized Institutional Review Board (CIRB reference 2017/2393). All methods were performed in accordance with the relevant guidelines and regulations. This study was funded by a grant from Mitsui Sumitomo Insurance Welfare Foundation. Omron Healthcare Singapore sponsored the Bio-Impedance Assessment (BIA) device.

## Results

The results of the 387 patients recruited for the baseline study were published [6]. Among these patients, 39 of them failed to be contacted or defaulted the subsequent review. A total of 348 patients had repeat anthropometric and clinical measurements, constituting a study adherence rate of 89.9%. The socio-demographic and clinical characteristics, physical activity, quality of life and anthropometry of the cohort of patients are reported in Table 1. At baseline, 26.1% of the subjects had sarcopenia while at follow up, the percentage increased to 35.9%.

**Table 1:** Demographic and clinical characteristics, physical activity level of the study population

	**Total, n (%)**	**Change in Muscle mass, Mean (SD)**	***p*-value**	**Change in Hand Grip Strength, Mean (SD)**	***p*-value**	**Change in Gait speed, Mean (SD)**	***p*-value**
**Total**	348 (100.0)	−0.67 (1.15)		−1.03 (4.61)		−0.04 (0.19)	
**Age, years**			0.562		0.959		0.529
**60–69**	218 (62.6)	−0.64 (1.16)		−1.04 (4.67)		−0.05 (0.18)	
**70 and above**	130 (37.4)	−0.72 (1.13)		−1.01 (4.53)		−0.03 (0.20)	
**Gender**			0.017		0.331		0.939
**Male**	184 (52.9)	−0.81 (1.15)		−1.26 (5.4)		−0.04 (0.19)	
**Female**	164 (47.1)	−0.52 (1.14)		−0.77 (3.52)		−0.04 (0.19)	
**Ethnicity**			0.630		0.958		0.500
**Chinese**	241 (69.3)	−0.65 (1.06)		−0.96 (4.68)		−0.03 (0.19)	
**Malay**	54 (15.5)	−0.84 (1.44)		−1.03 (4.52)		−0.05 (0.19)	
**Indian**	30 (8.6)	−0.64 (1.35)		−1.39 (4.25)		−0.03 (0.16)	
**Others**	23 (6.6)	−0.51 (1.11)		−1.28 (4.81)		−0.1 (0.18)	
**Marital status**			0.333		0.360		0.140
**Married**	290 (83.3)	−0.7 (1.20)		− 0.86 (4.85)		− 0.04 (0.18)	
**Single**	11 (3.2)	−0.68 (1.04)		− 0.87 (2.14)		−0.15 (0.17)	
**Divorced/Separated**	14 (4)	−0.91 (0.88)		−1.85 (4.26)		0.01 (0.25)	
**Widowed**	33 (9.5)	−0.34 (0.81)		−2.26 (2.75)		−0.02 (0.22)	
**Highest Qualification**			0.008		0.156		0.634
**Up to primary education**	82 (23.6)	−0.38 (1.18)		−0.4 (4.65)		−0.03 (0.19)	
**Secondary education and beyond**	266 (76.4)	−0.76 (1.13)		−1.23 (4.59)		−0.04 (0.19)	
**Type of dwelling**			0.541		0.790		0.595
**Public housing (Rental flat/1-3room)**	43 (12.4)	−0.13 (−2.66)		0.17 (−8.05)		−0.01 (− 0.41)	
**Public housing (4–5 room)**	259 (74.4)	−0.56 (−3.74)		− 0.54 (− 20.85)		− 0.02 (− 0.67)	
**Condominium/Private property**	46 (13.2)	−0.29 (−5.76)		0.76 (−13.80)		0.03 (−0.40)	
**Living**			0.837		0.150		0.615
**Alone**	15 (4.3)	−0.73 (0.82)		−2.71 (4.28)		−0.07 (0.18)	
**With family/ domestic helper**	333 (95.7)	−0.67 (1.16)		−0.95 (4.62)		−0.04 (0.19)	
**Comorbidities**							
**Hypertension/ High blood pressure**			0.756		0.262		0.367
Yes	302 (86.8)	−0.66 (1.14)		−1.14 (4.7)		− 0.04 (0.19)	
No	46 (13.2)	−0.72 (1.21)		−0.32 (3.97)		−0.02 (0.19)	
**Hyperlipidemia/ High Cholesterol**			0.805		0.814		0.557
Yes	335 (96.3)	−0.67 (1.16)		−1.04 (4.64)		−0.04 (0.19)	
No	13 (3.7)	−0.59 (0.93)		−0.73 (4.03)		−0.01 (0.16)	
**Ischemic Heart Disease**			0.553		0.589		0.761
Yes	64 (18.4)	−0.75 (1.1)		− 0.75 (5.48)		− 0.03 (0.19)	
No	283 (81.6)	−0.66 (1.16)		−1.1 (4.40)		−0.04 (0.19)	
	**Total, Mean (SD)**	**Change in Muscle mass, ρ**	***p*-value**	**Change in Hand Grip Strength, ρ**	***p*-value**	**Change in Gait speed, ρ**	***p*-value**
**Total number of comorbidities** ^**a**^	4.5 (1.6)	−0.038	0.480	− 0.030	0.584	0.009	0.873
**Total number of long term medications**	6.1 (2.5)	−0.045	0.403	−0.014	0.793	−0.009	0.869
**Follow up period (days)**	560 (122)	−0.358	< 0.001	0.064	0.237	−0.101	0.060
**Change from Baseline**							
**BMI**	0 (1.9)	0.153	0.004	0.039	0.472	0.002	0.964
**HbA1c**	−0.1 (1.0)	0.057	0.298	0.100	0.066	0.008	0.888
**PASE**	0.4 (88.4)	−0.045	0.408	0.088	0.105	−0.050	0.352
**WHOQOL**							
Domain 1	0.2 (1.8)	0.066	0.218	0.005	0.932	0.11	0.041
Domain 2	0.9 (1.8)	−0.013	0.806	0.062	0.247	0.018	0.744
Domain 3	2.2 (3.2)	0.128	0.017	−0.021	0.701	0.034	0.524
Domain 4	−0.5 (2.2)	0.109	0.042	0.020	0.714	−0.027	0.621
**IPAQ Total MET score**	− 347.5 (3510)	0.132	0.017	−0.035	0.527	−0.004	0.945

*PASE Physical Activity Scale for the Elderly, WHOQOL World Health Organization Quality of Life scale: Domain 1- Physical health, Domain 2-Psychological, Domain 3- Social relationship, Domain 4- Environment, IPAQ International Physical Activity Questionnaire*

^*a*^*Comorbidities refer to type-2 diabetes mellitus, hypertension, dyslipidemia, ischemic heart disease, transient ischemic attack, peripheral vascular disease*

Table 1: The mean age of the 348 patients was 68.4 ± 5.6 years. The majority were: males (52.9%), Chinese ethnicity (69.3%), married (83.3%), received secondary or higher education (76.4%), live in public housing (86.8%), live with family (95.7%), with at least one other non-communicable disease (96.3%), and had no change to their physical activity (52.9%) based on their IPAQ scores during the observation period. The change in the muscle mass was significantly associated with the male gender (− 0.81 vs − 0.52 kg/m^2^ in female, *p* = 0.017), secondary or higher education level (− 0.76 vs − 0.38 kg/m^2^, *p* = 0.008) in those with up to primary education, social relationship (*p* = 0.017) and environment (*p* = 0.042) domains in the WHOQOL scale. With increasing length of review interval, the change in muscle mass further declined (*r* = − 0.358, *p* < 0.001). Increase in BMI also increased the muscle mass change(*r* = 0.153, *p* = 0.004). Gait speed change was significantly associated with the WHOQOL physical health domain score (*p* = 0.041). No significant factor was significantly associated with the handgrip strength change.

Table 2: The mean change and standard deviation in muscle mass, hand grip strength and gait speed per month were − 0.03 (0.06) kg/m^2^/month, − 0.06 (0.26) kg/ month and − 0.002 (0.01) m/sec/month.

**Table 2:** Change in muscle mass, hand grip strength, and gait speed per month

	Mean (SD)	Median (IQR)
**Change in Muscle Mass (kg/m2/month)**	− 0.03 (0.06)	− 0.04 (− 0.07–0.01)
**Change in Hand Grip strength (kg/ month)**	− 0.06 (0.26)	− 0.05 (− 0.2–0.08)
**Change in Gait Speed (m/sec/month)**	−0.002 (0.01)	− 0.002 (− 0.008–0.003)

Table 3: The following clinical parameters are significantly reduced over the observation period: weight (− 0.49 ± 3.9 kg, *p* < 0.02), waist circumference (− 2.48 ± 5.96 cm, *p* < 0.001), hip circumference (− 3.18 + 5.09 cm, p < 0.001), systolic blood pressure (− 5.27 + 19.26 mmHg, *p* < 0.001), diastolic blood pressure (− 2.71 + 10.08 mmHg, *p* < 0.001), HBA1c (− 0.13 + 1.0%, *p* = 0.016), muscle mass change (− 0.67 + 1.15 kg/m^2^, *p* < 0.001),hand grip strength change (− 0.58 + 7.76 kg, *p* < 0.001) and gait speed change (− 0.04 (0.19), *p* < 0.001). No significant change is noted in mean PASE score. Their quality of life scores significantly increase in the psychological, social relationship and environmental domains in the WHOQOL scale.

**Table 3:** Change in clinical parameters of the study population between baseline and follow up visit

	Baseline	Follow up	***p***-value	Change from Baseline to follow up visit
Weight	65.7 (12.4)	65.3 (12.2)	0.020	−0.49 (3.90)
BMI	25.4 (4.1)	25.4 (4.2)	0.767	−0.03 (1.89)
Waist circumference	94.7 (10.3)	92.3 (10.2)	< 0.001	−2.48 (5.96)
Hip circumference	102.4 (8.1)	99.2 (8.1)	< 0.001	− 3.18 (5.09)
Systolic BP	140.8 (17.9)	135.5 (17)	< 0.001	−5.27 (19.26)
Diastolic BP	77.9 (9.8)	75.2 (9.7)	< 0.001	−2.71 (10.08)
HbA1c	7.2 (1)	7.1 (1.2)	0.016	−0.13 (1.00)
Total Cholesterol	4 (0.8)	4 (0.8)	0.510	0.03 (0.70)
HDL	1.3 (0.4)	1.3 (0.4)	0.987	0.00 (0.21)
LDL	2 (0.7)	2 (0.6)	0.533	0.02 (0.61)
TG	1.4 (0.7)	1.4 (0.6)	0.359	−0.03 (0.62)
Muscle Mass (kg/m2)	6.3 (1.3)	5.6 (1.5)	< 0.001	−0.67 (1.15)
Hand Grip strength (kg)	25.6 (8.1)	24.6 (8.1)	< 0.001	−0.58 (7.76)
Gait Speed (m/sec)	1.02 (0.17)	0.98 (0.2)	< 0.001	− 0.04 (0.19)
**PASE, Mean (SD)**	121 (80.6)	121.8 (82.8)	0.893	0.36 (88.45)
**WHOQoL**				
Domain 1	14.8 (1.7)	15 (1.4)	0.192	0.15 (1.82)
Domain 2	15.3 (1.8)	16.2 (1.3)	< 0.001	0.91 (1.85)
Domain 3	12.8 (3.1)	14.4 (2.4)	< 0.001	2.20 (3.19)
Domain 4	15.4 (1.9)	15.9 (1.1)	< 0.001	−0.48 (2.16)
**IPAQ Total MET score**	2624.4 (3043.2)	2276.9 (3327.3)	0.073	−347.5 (3510.3)

*PASE Physical Activity Scale for the Elderly, WHOQOL World Health Organization Quality of Life scale: Domain 1- Physical health, Domain 2-Psychological, Domain 3- Social relationship, Domain 4- Environment*

Table 4: With the potential factors (*p*-values <=0.2) included in the linear regression, the results show that lower education (*p* = 0.040), shorter length of review interval (*p* < 0.001),and increase in BMI (*p* = 0.001) were associated with increase of muscle mass change; while no change in medication class was associated with decrease of muscle mass change (*p* = 0.037); Singlehood (*p* = 0.033) and decline in the physical health (*p* = 0.038) based on WHOQOL scale were associated with gait speed reduction. No factor was significantly associated with change in hand grip strength.

**Table 4:** Factors affecting change in muscle mass, change in hand grip strength, and change in gait speed using linear regression

**Change in Muscle mass**	**Beta (95% CI)**	***p*-value**
**Gender**		
Male	− 0.19 (− 0.42–0.05)	0.117
Female	Ref	–
**Age**		
60–69	0.13 (− 0.11–0.37)	0.285
70 and above	Ref	–
**Highest Qualification**		
Up to primary education	0.36 (0.08–0.64)	0.012
Secondary education and beyond	Ref	–
**WHOQoL**		
Domain 3	0 (−0.04–0.04)	0.977
Domain 4	0.04 (−0.02–0.11)	0.187
**Length of review interval**	−0.003 (− 0.004--0.002)	< 0.001
**Change in BMI**	0.11 (0.05–0.17)	< 0.001
**Change in HbA1c**	0.1 (−0.01–0.22)	0.080
**Change in medication class**		
Increase in medication	0.39 (0.06–0.71)	0.019
Decrease in medication	−0.54 (−1.63–0.54)	0.327
No change	Ref	–
**Change in Insulin**		
Increase in insulin	0.19 (−0.35–0.72)	0.496
Decrease in insulin	0.17 (−0.55–0.89)	0.641
No change	Ref	–
**Change in medication dosage**		
Increase in dosage	−0.21 (− 0.52–0.11)	0.203
Decrease in dosage	−0.05 (− 0.41–0.32)	0.807
No change	Ref	–
**Change in Hand Grip Strength**	**Beta (95% CI)**	***p*-value**
**Gender**		
Male	−0.71 (−1.71–0.3)	0.167
Female	Ref	–
**Age**		
60–69	−0.16 (−1.17–0.84)	0.751
70 and above	Ref	–
**Highest Qualification**		
Up to primary education	0.81 (−0.38–1.99)	0.182
Secondary education and beyond	Ref	–
**Living**		
Alone	−2.01 (−4.35–0.33)	0.092
With family/ Domestic helper	Ref	–
**Change in PASE**	0 (0–0.01)	0.146
**Change in HbA1c**	0.45 (−0.04–0.94)	0.069
**Length of review interval**	0 (0–0.01)	0.148
**Change in medication class**		
Increase in medication	−1.19 (−2.54–0.16)	0.084
Decrease in medication	1.09 (−3.48–5.67)	0.639
No change	Ref	–
**Change in Insulin**		
Increase in insulin	0.47 (−1.77–2.72)	0.681
Decrease in insulin	−2.55 (−5.55–0.44)	0.094
No change	Ref	–
**Change in medication dosage**		
Increase in dosage	1.21 (−0.14–2.57)	0.079
Decrease in dosage	0.56 (−1.01–2.12)	0.485
No change	Ref	–
**Change in Gait speed**	**Beta (95% CI)**	***p*-value**
**Gender**		
Male	0.01 (−0.04–0.05)	0.812
Female	Ref	–
**Age**		
60–69	−0.01 (− 0.05–0.03)	0.575
70 and above	Ref	–
**Length of review interval**	0 (0–0)	0.607
**Change in HbA1c**	0 (−0.02–0.02)	0.720
**Marital status**		
Married	Ref	–
Single	−0.13 (−0.25--0.01)	0.029
Divorced/Separated	0.03 (−0.07–0.14)	0.515
Widowed	0.01 (−0.06–0.08)	0.713
**WHOQoL**		
Domain 1	0.01 (0–0.02)	0.024
**Change in medication class**		
Increase in medication	0.01 (−0.05–0.07)	0.732
Decrease in medication	−0.09 (− 0.28–0.1)	0.370
No change	Ref	–
**Change in Insulin**		
Increase in insulin	−0.01 (− 0.11–0.08)	0.791
Decrease in insulin	−0.02 (− 0.14–0.11)	0.807
No change	Ref	–
**Change in medication dosage**		
Increase in dosage	−0.03 (− 0.08–0.03)	0.376
Decrease in dosage	−0.07 (− 0.14--0.01)	0.033
No change	Ref	–

*WHOQOL World Health Organization Quality of Life scale: Domain 1- Physical health, Domain 2-Psychological, Domain 3- Social relationship, Domain 4- Environment*

## Discussion

The study tracks the anthropometric and muscle health parameters, together with self-reported physical activity and quality of life outcomes of older Asian patients with T2DM longitudinally over a period of up to 34 months. Hyperglycemia is a risk factor associated with sarcopenia compared with those with normoglycemia [13]. This study population of older patients had relatively stable glycemia and blood pressures based on their improved clinical outcomes in HbA1c, systolic and diastolic blood pressure indices (Table 1). Nonetheless, their muscle health parameters including muscle mass, strength and gait speed declined over the study period despite optimal control of their medical conditions and increased quality of life based on the WHOQOL scores (Table 3). The exception is the insignificant marginal QOL change in the WHOQOL physical health domain, which may indirectly reflect on the consequences of their gradual decline in muscle health (Table 3). Correspondingly, their weight, waist and hip circumferences decreased even though their self-reported physical activity level based on the PASE scores remained largely similar (Table 3). Overall, the results suggest regression of muscle health across the three major parameters over time in older patients with T2DM despite stable medical health status.

Males showed higher reduction in muscle mass compared with their female counterparts (Table 1) but gender becomes an insignificant factor after linear regression analysis (Table 4). Several studies have revealed a male preponderance towards sarcopenia based on AWGS criteria [14]. However, the study populations in these studies were heterogenous, comprising healthy volunteers or those with and without T2DM. Longitudinal analysis by Shimokata H et al. had shown that the skeletal muscle mass decreased with aging over a 12-year period except in Japanese middle-aged and elderly men [15]. However only 6.5% of men and 3.8% of women in this study population had T2DM. While insulin resistance is a risk factor associated with sarcopenia, its effect cuts across both gender in this study population with T2DM. Furthermore, the glycemic control remained stable over the study period.

Tables 1 and 4: The muscle mass of patients with lower education declined at a smaller magnitude compared to those with higher education level. Education was not associated with muscle strength and performance (Table 3). In contrast, the InCHIANTI study led by S Volpato et al. revealed that years of education were associated with reduced likelihood of sarcopenia in community dwelling older Italian people [16]. The years of education is a surrogate indicator for educational level, but with caveats. They also alluded to educational level being specifically correlated with muscle strength and walking speed rather than muscle mass alone. Nonetheless, they reported that their cross-sectional and observational design of the study limited any temporal or cause-effect relationship between muscle health parameters and associated factors. The older patients with lower education could have different lifestyle beyond physical activity. Potential confounding factors such as diet and nutrition status, were not recorded in this study, which constitute a limitation [17].

Table 4: Muscle mass decreases with time, which is compatible with ageing. It declines by an estimated mean of 0.003 kg/day or about 1 kg per year, alluding to a need for a review of muscle mass annually for any older person. Alternatively muscle mass increases by 0.1 kg with every unit increase in BMI (Table 4). BMI can be a surrogate marker for muscle mass if bio-impedance assessment is unavailable, assuming no other confounders on the BMI.

No significant demographic and clinical factor was associated with the change in grip strength. Hence, general evidence-based interventions can potentially alleviate the decline in muscle strength in older patients with T2DM, without targeting any specific risk factors. Muscle strength is a risk factor for adverse outcomes, such as falls, functional decline, cardiovascular disease and mortality in older people [18]. Its measurement is pivotal to determine the muscle health of any older person. However, monitoring of muscle strength based on grip strength using a dynamometer is not routinely conducted in primary care, as the device is often not available in most general practice clinics. The Singapore Multidisciplinary Consensus on Muscle Health recommends an alternative method of muscle strength by recording the timing to complete the 5-times Chair-Stand test [19]. The recommended cut-off is 8.5 seconds, which can be easily measured by a watch in a clinical setting. Such serial measurements of muscle strength can be used to motivate any older adult to take on suitable interventions to sustain their muscle health.

Muscle function or performance, as reflected in the gait speed, was significantly associated with physical health domain in the WHOQOL scale (Table 4). Mobility is determined by muscle performance [20]. In a local study of older people, mobility is the second commonest factor to impact on their quality of life [21]. Adequate muscle function enables the older people to ambulate and venture out of their residence to carry out their exercises and physical activities in the community. Aside from improving their physical health, their quality of life is also enhanced.

Singlehood is associated with lower muscle performance (Table 4). While the single older adult may live with other siblings in the same household, a proportion of them tend to stay alone or with a non-family room-mate due to local housing policy. In 2020, there were approximately 63.8 thousand single-person elderly households in Singapore [22]. Linton et al. have reported that older adults who live alone generally have comparable overall financial adequacy, physical and social well-being vis-à-vis to those who are in other living arrangements [23]. Nevertheless, a higher percentage of older adults living alone report having depressive symptoms, but it is uncertain if their symptoms are attributed entirely to their living arrangements [24]. Physical inactivity is associated with depressive symptoms, which may be detrimental to their muscle function. However, Linton et al. also reported that these depressive symptoms were moderated by the extent of the older persons’ social network. The authors also highlighted that the feeling of loneliness rather than the living arrangement per se which is associated with worse health outcome. Further research is needed to tease out the complex relationship between single older patients with T2DM and their muscle performance.

The PASE scores show insignificant change in physical activity among the older patients over the study period (Table 1). Their IPAQ scores showed that 52.9% of the study population had no change and 26.6% of them had decreased physical activities (Table 1). A local recent survey revealed similar physical activity profile of older adults with a median PASE score of 110, of which 37% of them spending eight or more sedentary hours daily [25]. Interventions are deemed necessary to strengthen their muscle health whilst keeping their glycaemia and other medical conditions under control. The Singapore Multidisciplinary Consensus Recommendations on Muscle Health suggests that for the uncomplicated cases, lifestyle modifications in exercise and diet can be initiated in the community without further assessment [19]. However, during the current COVID-19 pandemic, official enforcement measures such as isolation, quarantine and social distancing result in extended period of time at home. Consequently, reductions in physical activity and changes to dietary intake could potentially adversely affect the muscle health of these older people [26]. Home-based exercise is a potential solution to deter sedentary behavior and physical inactivity during the pandemic in older adults, especially those with T2DM in the study [27]. Suitable home-based physical activity and exercises will not only support their muscle health but also their glycemic control and mental health.

### Strength

This longitudinal study is one of the few which focuses on the short term clinical examination of muscle mass, strength and function in older patients with T2DM. The results show deterioration of muscle health despite stable glycemic control in this cohort of community dwelling Asian patients. Almost 90% of the study population had completed the study despite the current Covid-19 pandemic.

### Limitations

The study has its limitations. The framework for the muscle health parameters is based on the AWGS 2014 guidelines as the study was initiated in 2017 and continued till early 2021. The skeletal muscle mass is used as a major parameter instead of the appendicular muscle mass due to resources available for the study in 2017. The physical activity assessment is self-reported in the PASE and IPAQ scales, which is another limitation. Other factors such as dietary intake and nutritional status are not recorded in this study, which may confound the results. Another limitation is that we did not collect data on subjects who do not have diabetes for a fair comparison of their sarcopenia status to reflect the association between diabetes and sarcopenia.

## Conclusion

Despite stable glycemic control, older multi-ethnic Asian patients with T2DM had significant decline in their mean weight, waist and hip circumference, mean muscle mass and mean grip strength but no significant change in gait speed over a period of up to 34 months. Muscle mass change was associated with education level, BMI and length of review interval. No factor was significantly associated with grip strength. Gait speed was associated with singlehood and WHOQOL physical health domain. Preventive measures such as home-based physical activity may be a potential solution for this group of vulnerable patients, especially amidst the Covid-19 pandemic, but further research is needed to validate this option in strengthening their muscles.

## Data Availability

The datasets used and/or analysed during the current study available from the corresponding author on reasonable request.

## Abbreviations

AWGS: Asian Working Group for Sarcopenia
BMI: Body Mass Index
CI: Confidence Interval
CIRB: Centralized Institutional Review Board
DEXA: Dual-energy X-ray Absorptiometry
EMR: Electronic Medical Records
EWGSOP: European Working Group on Sarcopenia in Older People
HBA1c: Hemoglobin A1c/ Glycated Hemoglobin
HC: Hip Circumference
HDL: High-density Lipoprotein cholesterol
LDL: Low-density Lipoprotein cholesterol
NCD: Non-communicable disease
NEFA: Non-esterified Fatty Acids
OR: Odds Ratio
SD: Standard Deviation
SPSS: Statistical Package for Social Science
T2DM: Type 2 Diabetes Mellitus
TG: Triglycerides
WC: Waist Circumference
WHO: World Health Organization
